# Novel spontaneous myelodysplastic syndrome mouse model

**DOI:** 10.1002/ame2.12168

**Published:** 2021-05-14

**Authors:** Weisha Li, Lin Cao, Mengyuan Li, Xingjiu Yang, Wenlong Zhang, Zhiqi Song, Xinpei Wang, Lingyan Zhang, Grant Morahan, Chuan Qin, Ran Gao

**Affiliations:** ^1^ NHC Key Laboratory of Human Disease Comparative Medicine Beijing Engineering Research Center for Experimental Animal Models of Human Critical Diseases Institute of Laboratory Animal Sciences Chinese Academy of Medical Sciences (CAMS) and Comparative Medicine Center Peking Union Medical College (PUMC) Beijing China

**Keywords:** myelodysplastic syndrome (MDS), spontaneous mouse model, The Collaborative Cross Mice

## Abstract

**Background:**

Myelodysplastic syndrome (MDS) is a group of disorders involving hemopoietic dysfunction leading to leukemia. Although recently progress has been made in identifying underlying genetic mutations, many questions still remain. Animal models of MDS have been produced by introduction of specific mutations. However, there is no spontaneous mouse model of MDS, and an animal model to simulate natural MDS pathogenesis is urgently needed.

**Methods:**

In characterizing the genetically diverse mouse strains of the Collaborative Cross (CC) we observed that one, designated JUN, had abnormal hematological traits. This strain was thus further analyzed for phenotypic and pathological identification, comparing the changes in each cell population in peripheral blood and in bone marrow.

**Results:**

In a specific‐pathogen free environment, mice of the JUN strain are relatively thin, with healthy appearance. However, in a conventional environment, they become lethargic, develop wrinkled yellow hair, have loose and light stools, and are prone to infections. We found that the mice were cytopenic, which was due to abnormal differentiation of multipotent bone marrow progenitor cells. These are common characteristics of MDS.

**Conclusions:**

A mouse strain, JUN, was found displaying spontaneous myelodysplastic syndrome. This strain has the advantage over existing models in that it develops MDS spontaneously and is more similar to human MDS than genetically modified mouse models. JUN mice will be an important tool for pathogenesis research of MDS and for evaluation of new drugs and treatments.

## 1 INTRODUCTION

Myelodysplastic syndromes (MDS) form a group of heterogeneous myeloid clonal diseases characterized by abnormal differentiation and development of myeloid cells from hematopoietic stem cells,^1^, ^2^ and a high risk of conversion to acute myeloid leukemia (AML).^3^ The US National Cancer Institute first classified MDS as a tumor in 2001, and it is now considered as a common hematological tumor.^4^ Research on the physiological mechanisms and treatment of MDS still faces significant challenges. One of the main reasons is that most of the mutations that give rise to MDS cause disease with a short survival period, so patients usually do not survive to adulthood.^5^ Thus, research on the disease usually only focusses on the cellular level. Animal models are powerful tools for modeling and studying human diseases, and are very useful preclinical platforms for studying problems that cannot be easily (or at all) solved in the clinic.^6^ Current MDS models are usually produced by genetic modification (eg by introducing mutations as transgenes), by chemical induction, or by xenotransplantation.^6^, ^7^, ^8^, ^9^, ^10^ However, some of these models’ phenotypes cannot be maintained stably for a long time and some cannot simulate the pathogenesis of MDS from abnormal hematopoietic stem cells. MDS models induced experimentally can be quite different from the actual human clinical condition.^5^ A spontaneous MDS model would be more similar in onset to the human MDS process, and thus more helpful to translating research results to humans. Therefore there is an urgent need to establish a spontaneous animal model in order to analyze the pathogenesis of MDS and the process of transformation to AML.

The Collaborative Cross (CC) is a family of mouse strains produced by selective breeding from eight genetically diverse founder strains,^11^ and is the result of a project formally initiated in 2004 at The Jackson Laboratory. Each CC line originates from an independently breeding funnel so that every recombination site in the CC population is uniquely generated, and the CC strains harness the common genetic diversity of the mouse species,^11^, ^12^, ^13^ and can be used to identify genes mediating complex diseases and traits,^14^, ^15^ as well as providing models for human diseases, such as osteoporosis, diabetes complications, viral infections and a variety of spontaneous tumors.^16^, ^17^, ^18^, ^19^ We investigated a panel of CC strains^20^ and identified one, JUN, that appears to develop MDS spontaneously. As with the other CC strains, JUN was developed from an independently breeding funnel, and had genetic information of eight founder strains.

## 2 METHODS

### 2.1 Animals

JUN mice were provided by the Institute of Laboratory Animal Sciences, Chinese Academy of Medical Sciences, and housed in a specific pathogen‐free (SPF) and standard barrier environment with free access to food and water. The JUN mice bred up to 47 generations and 6 generations in our breeding environment. C57BL/6J mice purchased from Beijing Huafukang Bioscience company were used as controls. Both females and males had significant phenotypes, so the sex of the mice used in the study was random. All procedures in this study involving animals were reviewed and approved by the Institutional Animal Care and Use Committee of the Institute of Laboratory Animal Sciences, Chinese Academy of Medical Sciences & Peking Union Medical College (GR20004).

### 2.2 Blood samples

JUN mice (6‐8 weeks) were taken out of the SPF environment and maintained in a barrier environment for 1 month, and then compared to 10‐12 weeks old C57BL/6J mice. The mice were anesthesized by intraperitoneal injection of ketamine, xylazine, and benpiate hydrochloride (0.0375/0.0375/0.000125 mg/g body weight). Blood samples were then taken, stored in pediatric plastic tubes with the anticoagulant BDTM K_2_EDTA and analyzed using automated hematological analysis equipment (BAYER ADVIA 2120).

### 2.3 Mouse bone marrow cell isolation

Femurs and tibias were taken, and marrow cells were collected by flushing with PBS. The bone marrow cells were then analyzed by flow cytometry and blood smear.

### 2.4 Bone marrow and blood smear preparation

To prepare the blood smear, a drop of blood was taken from the mouse tail vein, dropped onto a glass slide, and then spread across the slide using another glass slide held at a 30° angle and pushed at a constant speed. The bone marrow smear was taken from the sternum of a mouse. The bone marrow cells in the sternum were squeezed onto a pre‐prepared glass slide, dripped with serum, and the droplet was spread across the slide in the same way as the blood smear. All smears were then stained with Wright‐Giemsa (Jiancheng Biotech, D010).

### 2.5 **Hematopoietic stem cell** **and progenitor cell analysis**

Following red blood cell lysis, bone marrow cells were stained with a cocktail of antibodies before analysis by flow cytometry. Lineage committed cells were identified as B220, CD19, CD93, CD43, CD24, CD34, CD16/32, CD135, c‐Kit, Sca1, and IgM (FITC, APC, PE, PerCP/Cy5.5, PE/Cy7, APC/Cy7 conjugated and from Biolegend) positive. Antibodies Sca1 (Biolegend) and cKit (Biolegend) are used to identify progenitor cell populations and hematopoietic stem and progenitor cell (HSPCs) (Lineage^−^, Sca1^+^, cKit^+^) and with antibodies CD16/CD32 (FcγRII/III) and CD34 (Biolegend) to distinguish common myeloid progenitors (CMP; Lineage^−^, Sca1^−^, cKit^+^, CD34^int^, FcγRII/III^int^), megakaryocyte‐erythrocyte progenitors (MEP; Lineage^−^, Sca1^−^, cKit^+^, CD34^lo^, FcγRII/III^lo^) and granulocyte‐macrophage progenitors (GMP; Lineage^−^, Sca1^−^, cKit^+^,CD34^int^, FcγRII/III^hi^), as well as with antibodies CD135 to show LT‐HSCs (CD135^−^, CD34^−^), ST‐HSCs (CD135^−^, CD34^+^) and multipotent progenitors (MPP; LSK, CD135^+^, CD34^+^). Sca1, ckit, CD19, B220, CD93, IgM, CD43, and CD24 were stained to determine the frequency of B‐cell precursor populations.

### 2.6 Immunohistochemistry

Hematoxylin‐eosin (H&E), CD34 (Abcam, ab81289), and MPO (Abcam, ab9535) stained sections from tissues such as thymus, lymph nodes, spleen, liver, kidney, lung, and tibia were evaluated using conventional staining techniques. Staining was performed on 5‐7 µmol/L slices cut from formalin‐fixed, paraffin‐embedded resected tissue. Photomicrographs were captured using the NDP.view.2 on an imaging analysis system (Nano Zoomer S60).

### 2.7 Flow cytometry

After red blood cell lysis, bone marrow cells for analysis by flow cytometry were stained with a cocktail of antibodies for 30 minutes. After isolation, cell suspensions were washed with 0.5% BSA in PBS and passed through 40 µm cell strainers (BD PharMingen), and cell populations were characterized by flow cytometry. Stained cells were analyzed separated by FACSAriaⅡ cell flow cytometer (BD Biosciences). Data were analyzed with FlowJo software.

### 2.8 Quantitative real‐time polymerase chain reaction (qRT‐PCR)

The mouse tissues were resected in a biosafety cabinet and then crushed after rapid freezing in liquid nitrogen. Total RNAs were isolated using TRIzol (Invitrogen, CA, USA) and cDNAs were obtained using a reverse transcription kit and the SYBR Green Master Mix kit (Takara, Otsu, Japan) following the manufacturer's protocol. The complementary DNA (cDNA) was amplified with the following primers: 5′‐CTGGTTACACTTGGGGTTGC‐3′ (forward) and 5′‐CCCCACCTGCCAATTTCTCA‐3′ (reverse) for RPS14; 5′‐AGGTCGGTGTGAACGGATTTG‐3′ (forward) and 5′‐TGTAGACCATGTAGTTGAGGTCA‐3′ (reverse) for GAPDH. Quantitative RT‐PCR was carried out using an ABI Prism 7900 Sequence detection system (BioRad CFX Connect). Relative levels were normalized to that of GAPDH.

### 2.9 Library construction and whole genome sequencing

A 1 μg sample of genomic DNA was randomly fragmented by Covaris. Fragmented DNA was selected using an Agencourt AMPure XP‐Medium kit to yield an average size of 200‐400 bp. The selected fragments were, through end‐repair, 3′ adenylated, adapter‐ligated, and PCR amplified and the products were recovered using the AxyPrep Mag PCR clean up Kit. An aliquote of the PCR products was taken for hybridization with BGI Hybridization and Wash kits. After that, the AxyPrep Mag PCR clean up Kit was used to recover the products as before. The double stranded PCR products were heat denatured and circularized using a splint oligo sequence to form single stranded circular DNA (ssCir DNA) as the final library, which was assessed by QC. The library was amplified to make DNA nanoballs (DNBs) which have more than 300 copies of one molecular. The DNBs were loaded into the patterned nanoarray and pair‐end 100 base reads were generated and sequenced by combinatorial Probe‐Anchor Synthesis (cPAS) on the BGISEQ‐500 platform (BGI‐shenzhen, China).

### 2.10 Statistical analysis

For each set of assays, three independent experiments were performed. Results are expressed as the means ± SEM. Statistical significance was calculated using the unpaired Student's *t* test. *P* values of less than .05 were considered significant. All tests were carried out using Prism software version 5 (GraphPad Software), and the gene sequencing data was analyzed using Integrative Genomics Viewer (IGV).

## 3 RESULTS

### 3.1 JUN mice have a hematological disease‐like phenotype

**FIGURE 1:**
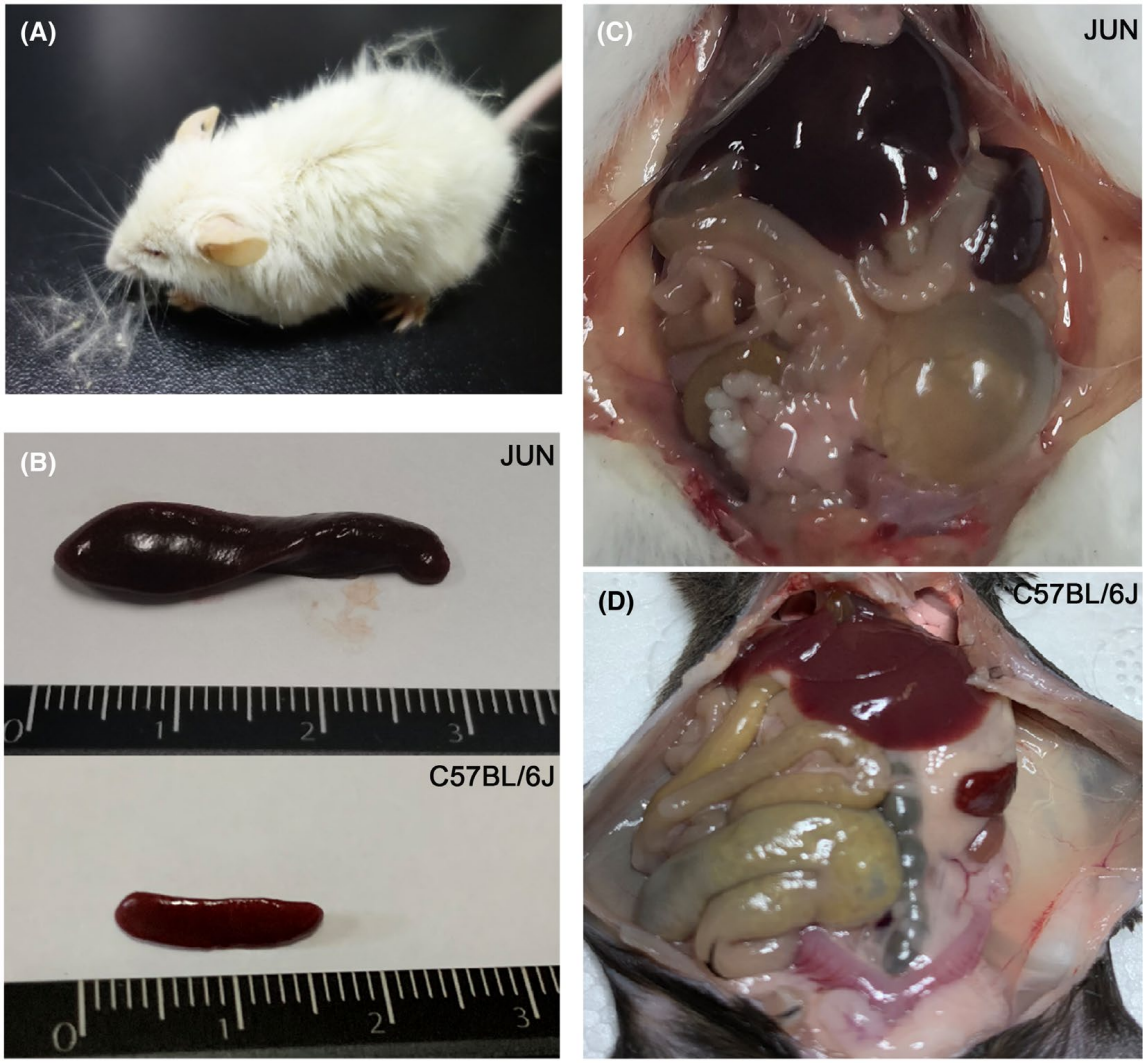
Phenotype of JUN mice. A, The appearance of the mice after living in a non‐SPF environment for 2 mo, showing changes in coat color and low vitality. B, Spleens of JUN mice that died naturally in a non‐SPF environment, showing obvious enlargement compared to C57BL/6J mice. C, Anatomy of JUN mice. D, Anatomy of normal mice C57BL/6J

Most mice of the albino JUN strain grew well in the SPF environment, having a smooth coat and lively character. However, the life span of all JUN mice was shorter than that of C57BL/6J mice, with about 10% of JUN mice showing ascites symptoms and large bellies when they were about 8 months old. Moreover, several months after the 6‐ to 8‐week‐old JUN mice were transferred to a non‐SPF environment, their coats become wrinkled and yellow, they become lethargic, and some died quickly (Figure 1A). We simultaneously transferred JUN and C57BL/6J and other two CC strains, NUK and LOT, into the same non‐SPF environment, and only the JUN mice showed the above symptoms. Our observations showed that the JUN mice become lethargic after 2 months, and after 3 months they were obviously listless and their feces were thin and yellow. Half of them died after 6 months, and the longest survival time of JUN mice after being moved to a non‐SPF environment was 9 months. On post‐mortem, splenic enlargement was observed (Figure 1B), and the liver was dark red and slightly enlarged compared with normal C57BL/6J mice (Figure 1C,D). Most of the content of the gastrointestinal tract was yellow fluid (Figure 1C,D). Pathological findings from the dead mice suggested that they had acute myelogenous leukemia. The observation of splenomegaly is also a sign of hematopoietic diseases.^21^ The unfavorable phenotype of the JUN mice is similar to the susceptibility to infection of patients with blood diseases,^22^ so we initially thought that JUN mice had spontaneous myelocytic leukemia.

### 3.2 Lesions in multiple organs of JUN mice

**FIGURE 2:**
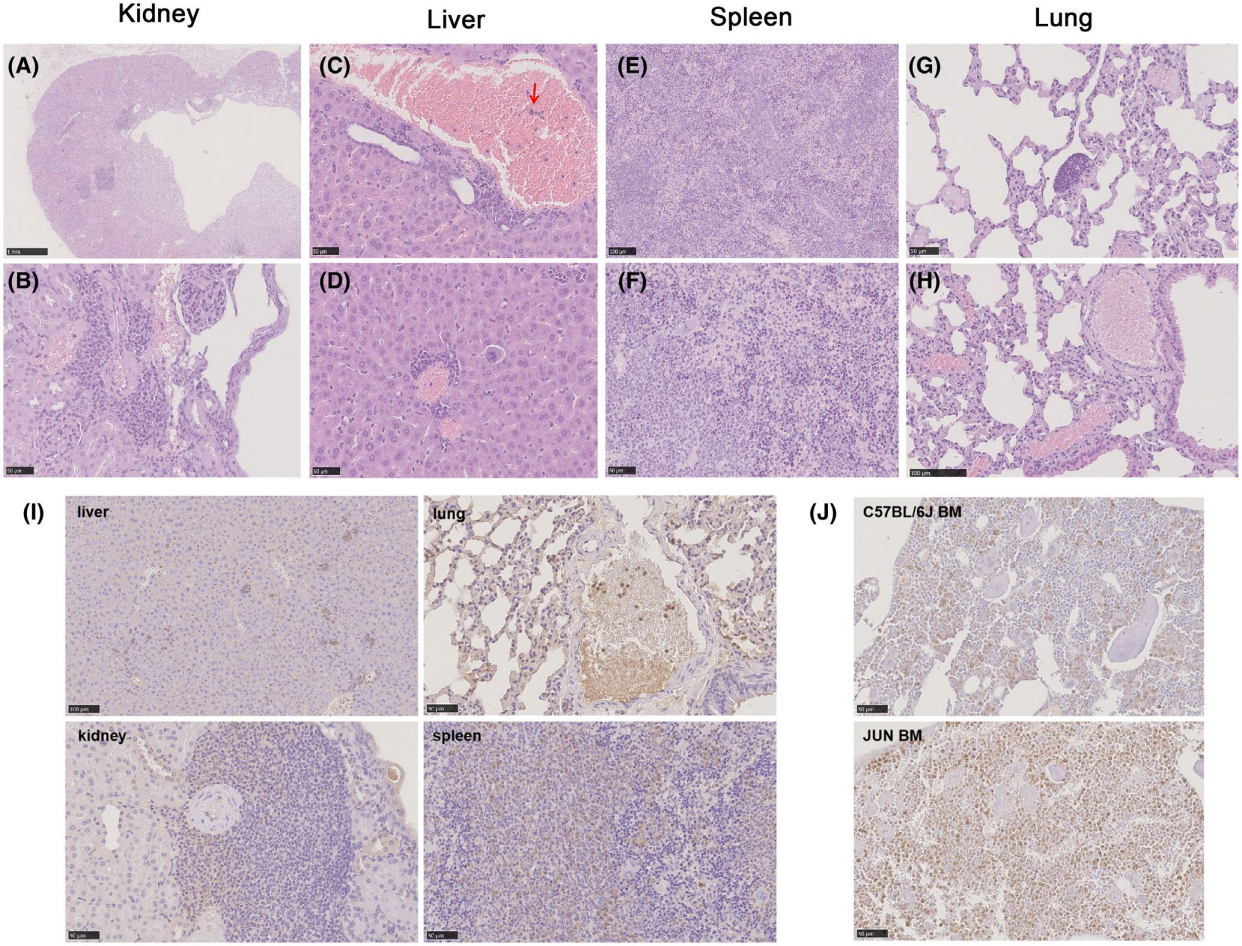
Histopathological changes in kidney, liver, spleen, and lung tissues Panels A‐H show representative tissues stained with Hematoxylin & Eosin (H&E, ×40). Red arrows indicate infiltration of basophilic premature mononuclear cells in vessels. I, Sections of JUN liver, spleen, lung, and kidney were stained with anti‐MPO antibodies. J, Compared with C57BL/6J, cells in bone marrow stained with anti‐MPO were significantly increased (scale bars = 50 µm)

Histological analysis was conducted on various organs of JUN mice that had died naturally after living in a non‐SPF environment. Lesions were identified in the kidney, liver, spleen, and bone marrow. Kidneys showed hydronephropathy, with quite a few basophilic mononuclear cells infiltrated into the renal interstitium (Figure 2A,B). Spleens showed severe splenic increased extra‐medullary hematopoiesis, with small nests of hematopoietic cells in the red pulp with difficult‐to‐define borders. Other hyperplastic lesions in the spleen included an abnormal increase and scattered infiltration of lymphoid and erythroid cells (Figure 2E,F). Furthermore, the liver also exhibited extra‐medullary hematopoiesis with random infiltrating basophilic mononuclear lymphoid cells (Figure 2C,D). Vessels in the pulmonary parenchyma (Figure 2G,H) and other organs also showed basophilic premature mononuclear cell infiltration (Figure 2B‐D). Here, high levels of myeloperoxidase (MPO), a marker of both benign and neoplastic myeloid cells, were observed in many organs (Figure 2I,J).^23^, ^24^ Individual cells scattered throughout the liver and spleen were labeled; many were within areas that appeared to be myeloid hematopoietic. This was consistent with our initial suspicions that these JUN mice had myeloid leukemia.

### 3.3 Abnormal development of white blood cells in JUN mice

**FIGURE 3:**
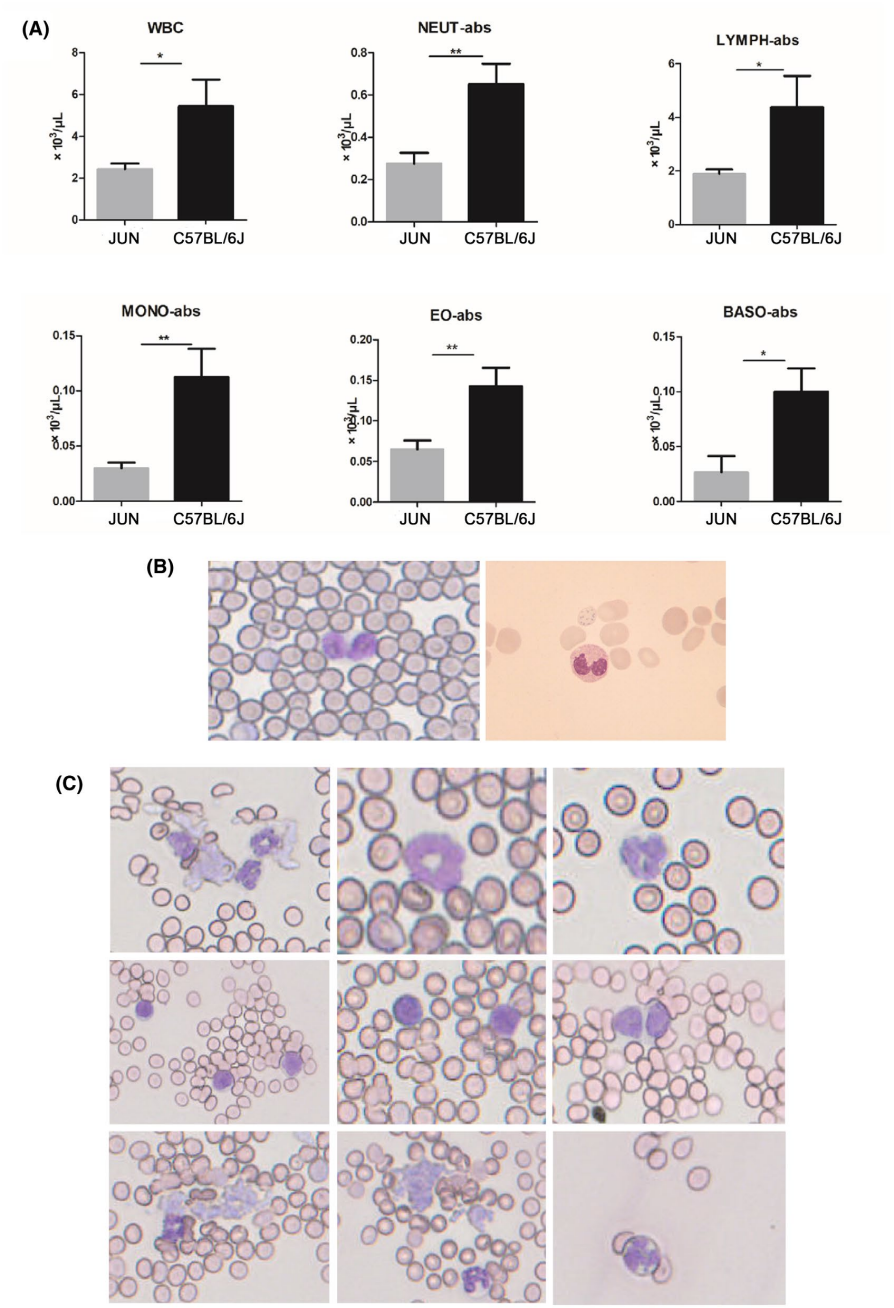
Hematological analyses. A, Peripheral blood counts of JUN mice (n = 5, two females and three males) and C57BL/6J (n = 4, mice, two females and two males). The data are presented as means ± SEM, ns: no significant difference, *P < .05, **P < .01. B, Comparison of Pseudo‐Pelger‐Hüet cells found in mouse (left) and human MDS patient's (right) peripheral blood smears. C, Abnormal morphology of white blood cells in peripheral blood smears of JUN mice. Mouse blood smears were prepared by Giemsa staining, and human blood smears were obtained from the Hematology Department of Peking University People's Hospital

To determine whether the JUN mouse strain progresses to myeloid leukemia, samples were collected for hematological analysis from 14‐week‐old mice that had lived in the non‐SPF environment for 2 months. WBC counts were lower in JUN mice compared with C57/BL6 mice, manifesting as cytopenia. The lower counts were generally due to lower numbers of neutrophils, monocytes, and lymphocytes (Figure 3A). Although the loss of WBCs does not affect the diagnosis of myeloid leukemia, we also found Pseudo‐Pelger‐Hüet cells in JUN blood (Figure 3B); these cells are often seen in MDS.^25^ Furthermore, in peripheral blood smears, we also found the abnormal development of neutrophils (Figure 3C), which is a major criterion in the assessment of dysplasia in MDS.^25^ Evaluation of the number and morphology of blasts in peripheral blood is the only peripheral blood parameter incorporated into the WHO classification of MDS, and is therefore critical.^26^ Firstly, we found that immature leukocytes accounted for an increased proportion of leukocytes (Figure 3A); the next most abundant blast cells observed in peripheral blood were immature granulocytes, circulating blast cells, and platelet clusters (Figure 3C). It is well known that MDS can transform into acute myeloid leukemia. In the initial period, these mice showed no any abnormalities, so, combined with phenotype and pathological analysis, it was concluded that JUN mice are likely to have MDS, with later progression to acute myeloid leukemia.^25^, ^27^

### 3.4 Abnormal differentiation of progenitor cells in the bone marrow of JUN mice

**FIGURE 4:**
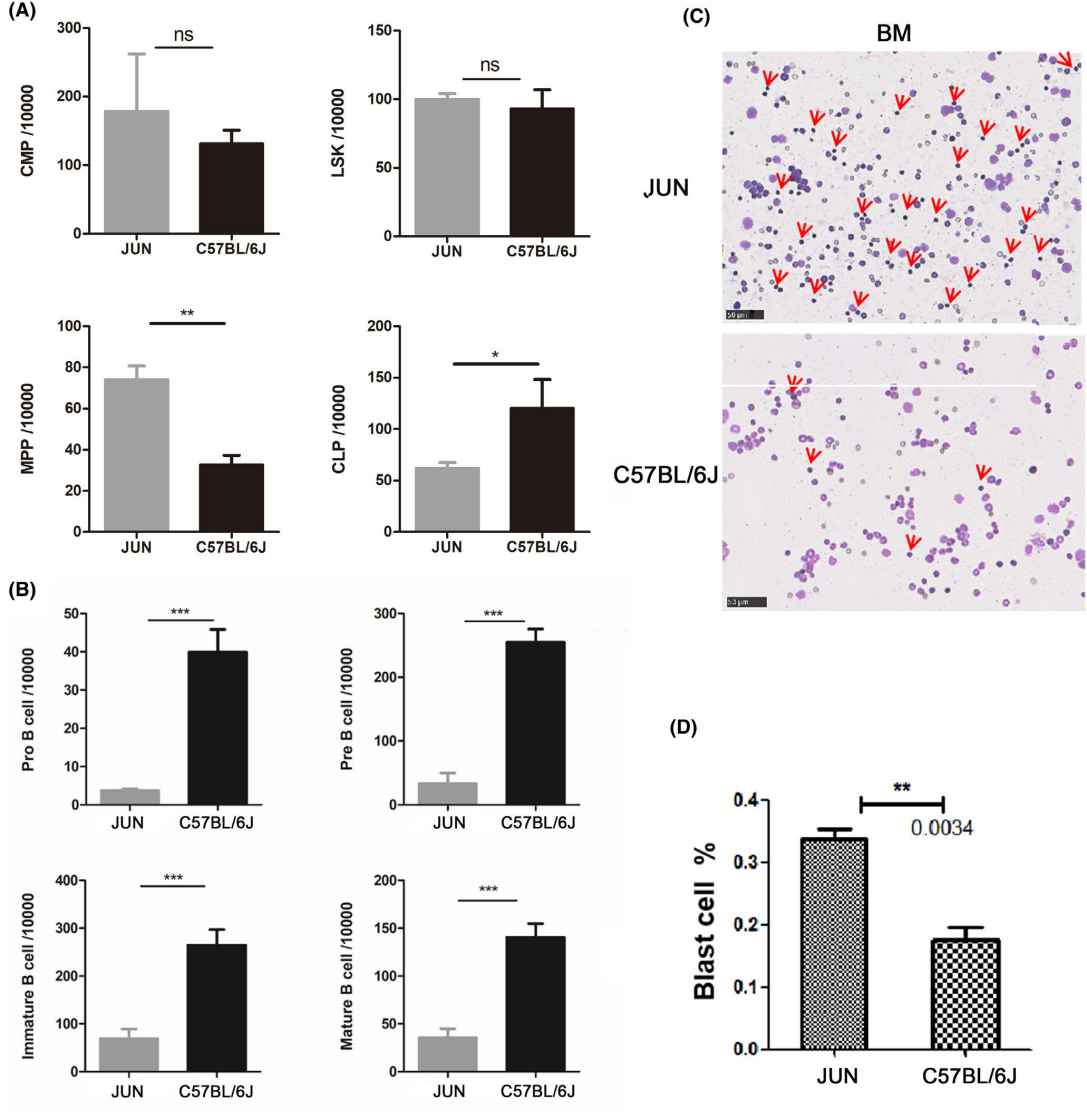
Analyses of JUN and MDS human bone marrow cells. Panels A and B show sort and count of cells from bone marrow by flow cytometry. A, Count statistics of LSK (Lin^−^/Sca1^+^/c‐Kit^+^), CMP (Lineage^−^, Sca1^−^, cKit^+^, CD34^int^, FcγRII/III^int^) and MPP (LSK, CD135^+^, CD34^+^) cells of JUN (n = 4, 2 females and 3 males) and C57BL/6J (n = 4, 2 females and 2 males) mice. B, Count statistics of pro‐B‐cells, pre‐B‐cells, immature B‐cells and mature B‐cells of JUN (n = 5, 2 females and 3 males) and C57BL/6J (n = 4, 2 females and 2 males) mice. C, In bone marrow smears, compared with C57BL/6J, the number of abnormal megakaryocytes in JUN mice increased significantly. D, Count of abnormal blasts in bone marrow smears of JUN and C57BL/6J mice (n = 5, 2 females and 3 males). Results are presented as means ± SEM, ns: no significance, *P < .05, **P < .01, ***P < .001

Femoral bone marrow cells were extracted from JUN mice that had been maintained in the non‐SPF environment for a period of time. The bone marrow cells were classified and counted by flow cytometry. There was a significantly higher number of multipotent progenitor (MPPs) cells in the bone marrow of JUN mice compared to normal C57BL/6J mice, and the number of common lymphoid progenitor (CLP) cells, which can differentiate and develop into T cells, B nucleus and NK cells, was also reduced in JUN mice (Figure 4A). Although it is still controversial whether non‐myeloid lineages are involved in MDS, some cases have demonstrated B cell involvement.^28^, ^29^, ^30^ In our study, the differentiation of B cells from MPPs was blocked (Figure 4B). In addition, bone marrow smears showed a significant increase in the number of abnormal‐appearing loosely scattered blasts, (Figure 4C,D). As is well known, MDS is a group of heterogeneous clonal tumors caused by the aberrant development of hematopoietic stem cells.^31^ JUN mice showed abnormal differentiation of MPPs, leading to cytopenia in peripheral blood. This raised the possibility that abnormal development of hematopoietic stem cells causes spontaneous MDS in mice, which eventually progresses to AML.

### 3.5 Nuclear abnormalities were found in JUN bone marrow cells

**FIGURE 5:**
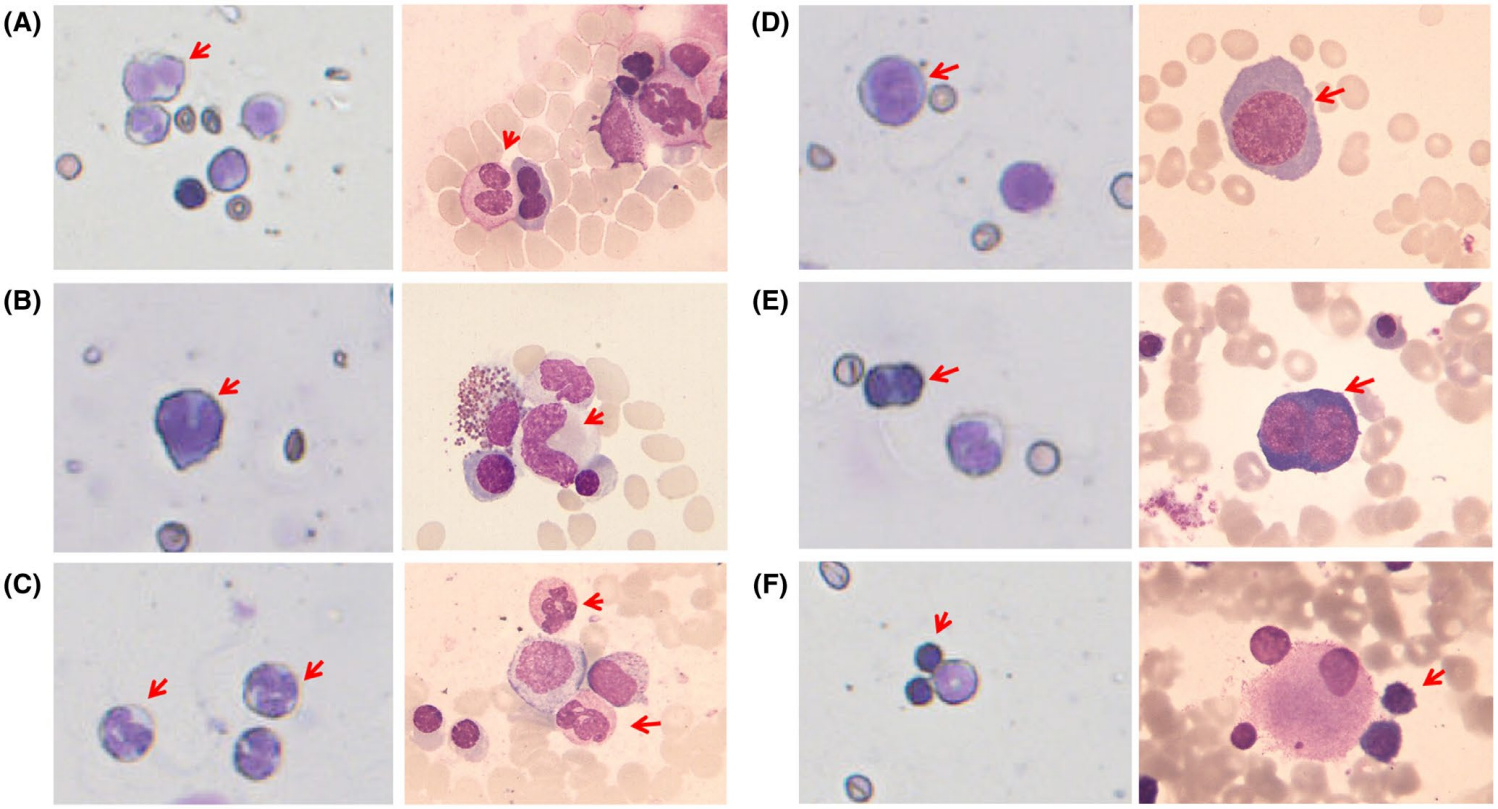
Comparative analysis of cell morphologies in bone marrow of JUN mice (left; magnification 40×) and human MDS patients (right; magnification 100×). A, Pseudo‐Pelger‐Hüet cells, (B) stab granulocytes, (C) segmented form granulocytes, (D and E) megaloblastoid changes, (F) micromegakaryocytes

To obtain more evidence that JUN mice suffer from MDS similar to human diseases, an appropriate morphologic assessment of dysplasia in bone marrow smears was essential, as this is a diagnostic criterion of MDS.^32^ The diagnosis of MDS can also be established in a markedly cytopenic mouse if a typical (MDS‐related) nuclear anomaly is found.^33^, ^34^ Therefore, we compared JUN cells with human bone marrow smears collected from the hematology of Peking University People's Hospital. It was found that the characteristic abnormal nuclear abnormalities observed in human MDS bone marrow, including Pseudo‐Pelger‐Hüet cells (Figure 5A), stab granulocytes (Figure 5B), segmented form granulocytes (Figure 5C), megaloblastic changes (Figure 5D,E), and micromegakaryocytes (Figure 5F), were also found in JUN.

### 3.6 A high CD34^+^ cell count has been found in JUN mice

**FIGURE 6:**
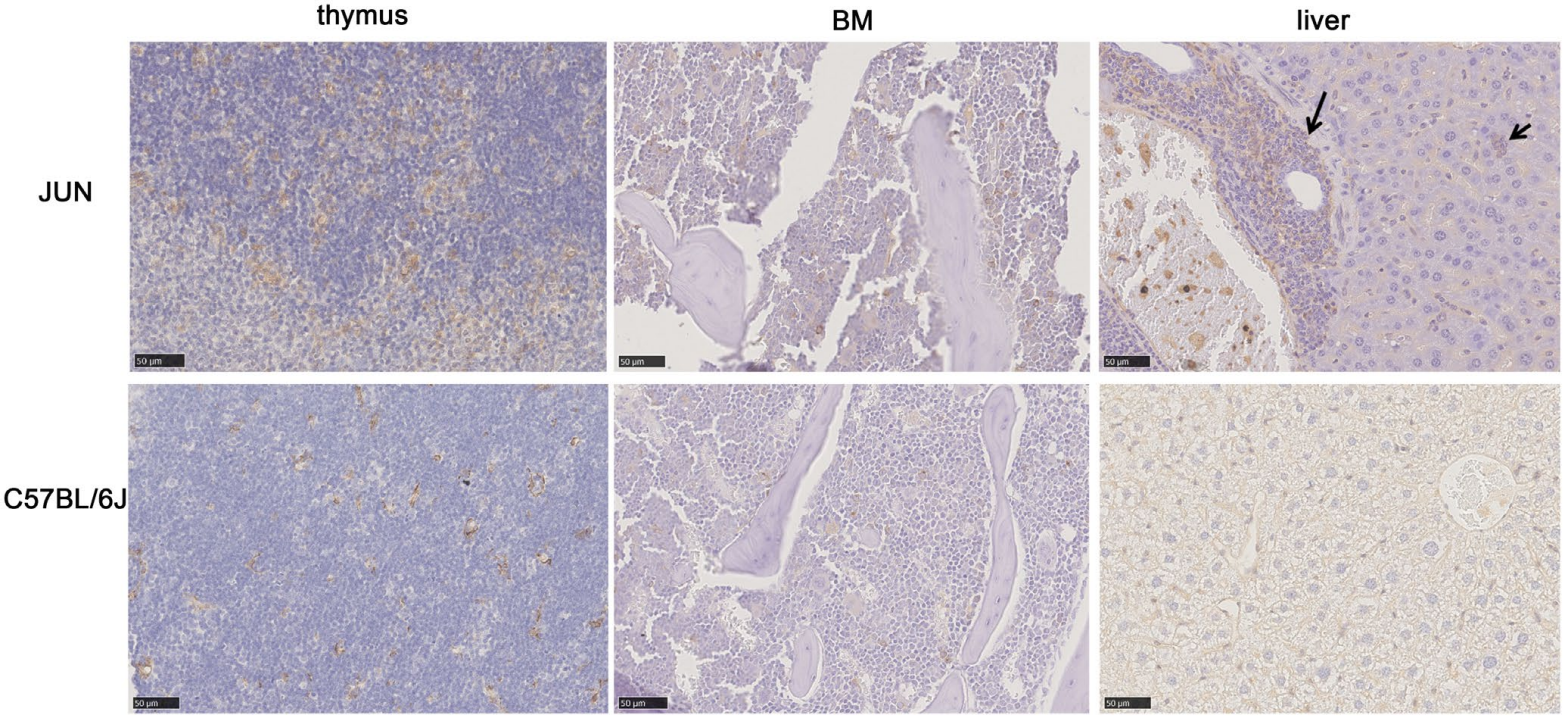
Representative images of anti‐CD34 immunohistochemistry staining. The stained organs are the thymus, bone marrow and liver of JUN and C57BL/6J mice. (Scale bar = 50 μm)

Currently, there is little consensus as to which cell type should be defined as an MDS “stem cell”. At present, CD34^+^ cells possessing a clonal marker comprise the best prototype.^35^, ^36^ Antibodies against CD34 help to estimate the number of CD34^+^ progenitor/blast cells and to detect small blast cell infiltrates indicative of progression/transformation to AML.^32^ Here, we used anti‐CD34 antibodies to analyze early MDS stem/progenitor cells. The bone marrow showed a slight increase in CD34^+^ blast cells. In JUN thymus, CD34 immunohistochemistry revealed an obvious increase in blast cells, ranging between 10% and 20%, with these cells showing a slight tendency to congregate. Even the liver had a significant increase in CD34^+^ blast cells (Figure 6). This result further supports the possibility that spontaneous MDS in JUN mice can develop into AML.

### 3.7 JUN mice have a mutation in the *Rps14* gene

**FIGURE 7:**
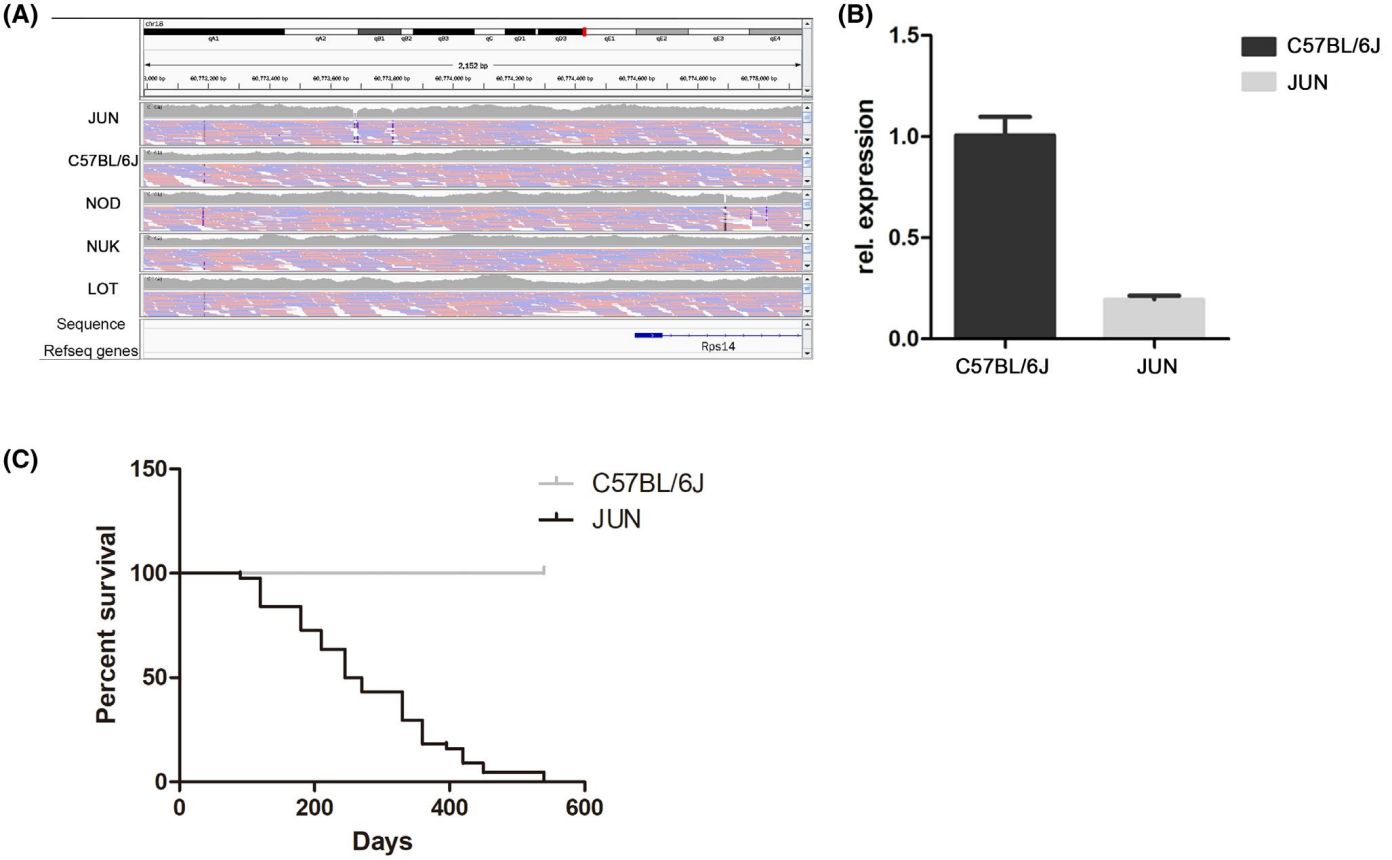
JUN mice have a mutation in the *Rps14* gene. A, Comparative analysis results of the gene sequencing. Only JUN has a mutation in *Rps14* gene. Both deletion and insertion sites were located between 60773600 bp and 60773700 bp in the qA2 region of chromosome 18; gray shows coverage, black means there is a deletion, and blue indicates inserts with fragments. B, *Rps14* expression in the spleen of JUN and C57BL/6J was determined by RT‐PCR. C, Survival curve of JUN mice (n = 44)

Finally, we did DNA sequencing in peripheral blood, and analyzed MDS related mutant genes, comparing JUN with two of its founder strains (C57BL/6J and NOD) and two other CC strains (NUK and LOT), and found that there was a deletion and an insertion mutation of the *Rps14* gene in the qA2 region of chromosome 18. Querying the Sanger Mouse Genomes Project^37^ database showed that none of the other CC founder strains have this deletion, nor do they have any missense mutations in *Rps14*. Significantly, the expression of *Rps14 i*n spleen was also reduced in JUN compared with C57BL/6J (Figure 7B). The severe macrocytic anemia in del(5q) MDS patients has been linked to haploinsufficiency of (*Rps14*),^38^, ^39^ and it has been described in CD34^+^ cells.^40^ Based on this, some conditional *Rps14* knockout MDS models have been generated, that have developed a progressive anemia.^39^ Our research found that, unlike the conditional *Rps14* knockout MDS models whose *Rps14* was excised in hematopoietic cells, *Rps14* was not missing in JUN mice but the expression level was significantly decreased. Moreover, these two models showed different MDS phenotypes: JUN mice mainly showed leukopenia, while the reticulocyte count of *Rps14* haploinsufficient mice decreased precipitously. Therefore, it is likely that the mutation of *Rps14* is one of the factors contributing to the spontaneous MDS exhibited by the JUN strain.

Finally, our analysis of all dead Jun mice found an 86% probability of progression to AML. A survival curve for JUN mice was calculated and showed that the average life span was about 279 days (Figure 7C). The mice used for laboratory tests all showed corresponding pathological characteristics of MDS, and most of them could have progressed to AML.

## 4 DISCUSSION

Currently, the treatment methods for MDS are stem cell transplantation, drug therapy, and supportive care.^3^, ^41^ Allogeneic hematopoietic stem (progenitor) cell transplantation (HSCT) is considered as the only potentially curative therapy for MDS.^42^ However, these different treatments for MDS patients are disappointing, because positive response rates are low and responses are often not sustained.^2^, ^43^

**FIGURE 8:**
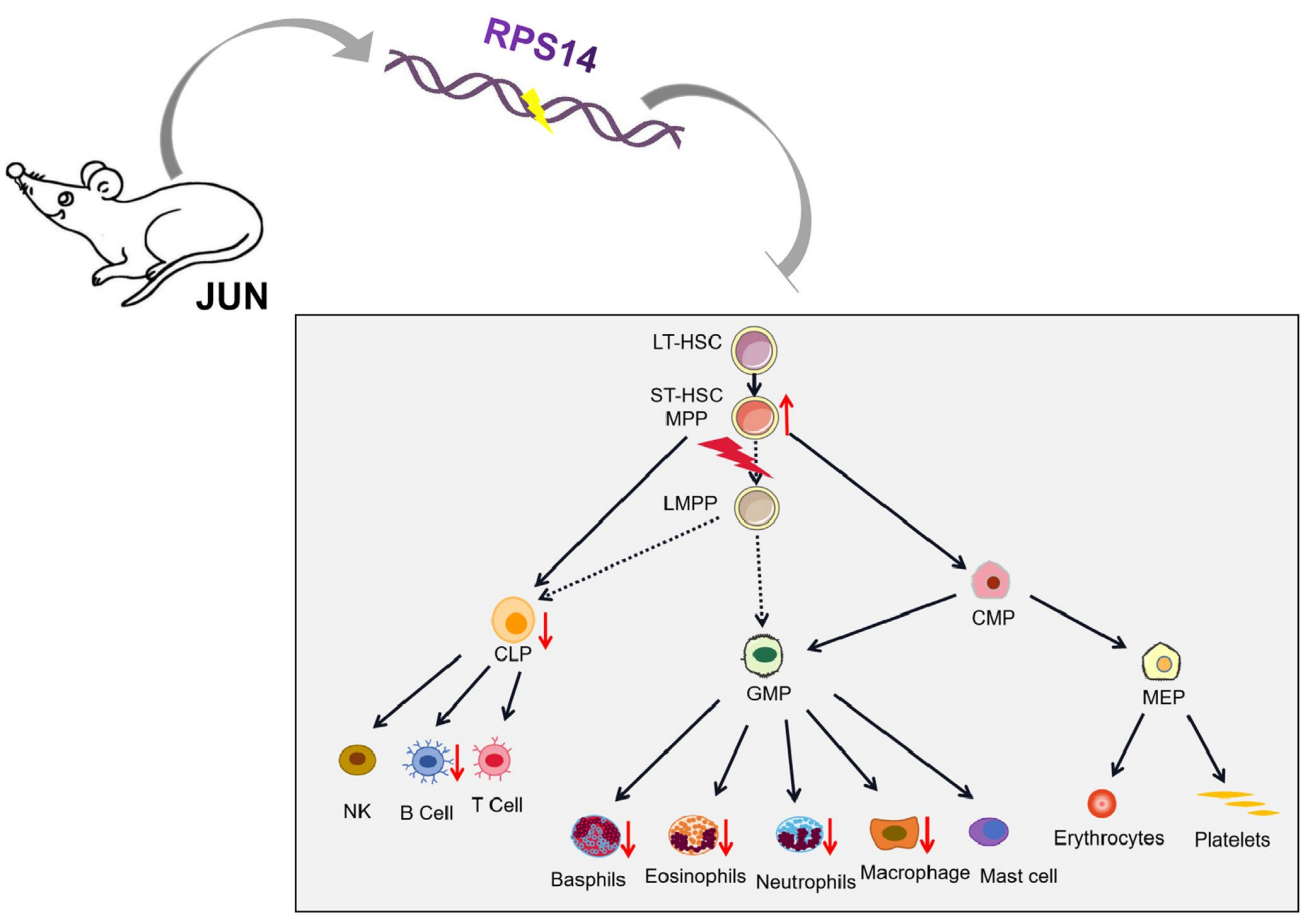
Summary of our findings on JUN mice with MDS. The mutation of the *RPS14* gene in JUN mice may lead to the abnormal differentiation of MPP, followed by the symptoms of hemopenia

Mouse models can provide a platform for pre‐clinical testing, including testing novel small molecules (singly or in combination) and can even be used to study how HSCT works for patients with MDS. CC mice whose phenotypic diversity is on a par with human populations have proved very useful for providing novel animal models of human disease.^16^ By combining the MDS diagnosis in mice suggested by the Mouse Models of Human Cancers Consortium (MMHCC) and human MDS suggested by the World Health Organization (WHO),^31^, ^44^ we diagnosed cytopenia in peripheral blood of JUN mice but no leukocytosis or erythrocytosis. There were lower levels of neutrophils, basophils, eosinophils, monocytes, and lymphocytes in the peripheral blood, and the reason for this was dysplasia of bone marrow cells. Moreover, we showed that the abnormally developed cells in bone marrow were MPPs (Figure 8). In subsequent experiments, we also verified that the abnormal development of cells in JUN mice had a myeloid origin by detecting CD34^+^ cells and MPO positive cells and found MDS related mutations in the *Rps14* gene. Therefore, we concluded that JUN mice suffer from MDS and which can develop into AML.

1. JUN mice with spontaneous MDS provide an exciting and promising model for studying the mechanisms both of MDS development and of conversion of MDS into AML. This spontaneous MDS model can help facilitate the development of new drugs and pre‐clinical evaluation of new therapeutic approaches. Agents in development for MDS in clinical trials, such as kinase inhibitors, deacetylase inhibitors and DNA methyltransferase inhibitors can be tested in the spontaneous model to ensure efficacy before recruiting patients.^45^, ^46^ By testing in JUN mice, the mechanism of drugs currently used can be clarified, to provide a deeper understanding of the underlying mechanisms in heterogeneous diseases, and hopefully improve our ability to treat MDS patients. Due to the high spontaneous incidence of MDS in JUN mice and the high frequency of conversion to AML, studies using these mice would be more stable and reliable than other models. In addition, further study of the *Rps14* gene to explore its role in the hematopoietic system would be of great interest.

## CONFLICT OF INTEREST

The authors declared no conflict of interest.

## AUTHOR CONTRIBUTIONS

The JUN mice were bred by Grant Morahan and Lingyan Zhang; Weisha Li and Ran Gao conceived and designed the study; Weisha Li, Xingjiu Yang and Mengyuan Li performed the experiments. Weisha Li, Lin Cao, Wenlong Zhang, Zhiqi Song and Xinpei Wang collected and analyzed the data. Grant Morahan provided the JUN genomic sequences. Weisha Li wrote the original draft of the manuscript, Ran Gao, Wenglong Zhang and Grant Morahan revised the manuscript. All authors critically read and contributed to the manuscript and approved the final version.
